# Angiotensin type-2 (AT-2)-receptor activation reduces renal fibrosis in cyclosporine nephropathy: evidence for blood pressure independent effect

**DOI:** 10.1042/BSR20160278

**Published:** 2016-11-03

**Authors:** Giovanna Castoldi, Cira R.T. di Gioia, Raffaella Carletti, Francesca Roma, Gianpaolo Zerbini, Andrea Stella

**Affiliations:** * Clinica Nefrologica, Ospedale San Gerardo di Monza, Dipartimento di Medicina e Chirugia, Università degli Studi di Milano-Bicocca, Monza 20900, Italy; † Dipartimento di Scienze Radiologiche, Oncologiche e Anatomopatologiche, Istituto di Anatomia Patologica, Sapienza Universita’ di Roma, Roma 00161, Italy; ‡ Unita' Complicanze del Diabete, Ospedale San Raffaele, Milano 20132, Italy

**Keywords:** AT-2 receptors, compound 21, cyclosporine nephropathy, experimental models, myocardial fibrosis, rats, renal fibrosis

## Abstract

Compound 21 (C21), selective agonist of angiotensin type-2 (AT-2) receptors, shows anti-inflammatory effects in experimental models of hypertension and nephroprotection in diabetes. The aim of the present study was to evaluate the effects of C21 in cyclosporine nephropathy, which is characterized mainly by tubulo-interstitial fibrosis. Ten days before and during the experimental periods, low-salt diet was administered to Sprague–Dawley rats. Cyclosporine-A (CsA; 15 mg/kg per day, intraperitoneal injection) and CsA plus C21 (0.3 mg/kg per day, intraperitoneal injection) were administered for 1 and 4 weeks. Control groups were left without any treatment. Blood pressure (plethysmographic method) and 24 h urinary albumin excretion were measured once a week. At the end of the experimental protocols, the kidneys were excised for histomorphometric analysis of renal fibrosis and for immunohistochemical evaluation of inflammatory infiltrates and type I and type IV collagen expression. After 1 and 4 weeks, the rats treated with CsA showed a significant increase (*P*<0.01) in blood pressure, no significant changes in urinary albumin excretion and a significant increase (*P*<0.01) in glomerular and tubulo-interstitial fibrosis and inflammatory infiltrates as compared with the control rats. Treatment with C21 did not modify the CsA dependent increase of blood pressure, which was higher than in control rats, but after 4 weeks of treatment significantly reduced (*P*<0.01) glomerular and tubulo-interstitial fibrosis, type 1 collagen expression and macrophage infiltration, as compared with rats treated with cyclosporine. The administration of C21 showed a protective effect on cyclosporine nephropathy, decreasing renal fibrosis and macrophage infiltration. These data suggest that C21 may counteract tubulo-interstitial fibrosis, the most potent predictor of the progression of renal diseases.

## INTRODUCTION

Renal fibrosis represents a serious complication of chronic kidney diseases, leading to end-stage renal failure, independently on the aetiology of initial renal injury [1].

The increase in collagen and extracellular matrix protein deposition in glomerular and tubulo-interstitial area, modulated by multiple molecular pathways, characterizes the progression of renal fibrosis [2,3].

Generally, in the development of renal fibrosis it is recognized an initial increase in the synthesis of growth factors and cytokines by resident cells, accompanied by the influx of inflammatory cells, monocyte-macrophages, which further promotes the release of cytokines and matrix proteins [3]. These alterations are followed by an excessive deposition of extracellular matrix proteins and by an abnormal process of matrix remodelling, which leads to the formation of renal scarring and finally to organ failure [4].

Despite the evidence that the fibrotic processes may affect all the renal districts, tubulo-interstitial fibrosis represents the most potent predictor of outcome in renal diseases [5,6].

Cyclosporine-A (CsA) is a calcineurin inhibitor widely used for many years in organ transplantation [7]. CsA treatment has improved the survival in transplanted patients, but its use is burdened by side effects such as hypertension and nephrotoxicity [8–11]. Immunosuppressive medications, particularly CsA treatment, are associated with post-transplant hypertension, which is a common clinical problem in transplanted patients [12], increasing cardiovascular mortality and morbility.

Chronic CsA nephropathy is a major cause of chronic allograft dysfunction and allograft failure in renal transplant recipients [8] and is characterized by tubular atrophy, inflammatory cell infiltration and hyalinosis of the afferent arterioles, followed by the development of tubulo-interstitial fibrosis.

Renin–angiotensin–aldosterone system is an important regulator of renal and myocardial fibrosis, and drugs interfering with the renin–angiotensin–aldosterone system, as angiotensin converting enzyme (ACE) inhibitors and angiotensin type 1 receptor blockers, presently represent the tools of choice to counteract the progression of renal and cardiovascular diseases [13].

Angiotensin II (Ang II), the main effector of the renin–angiotensin system, binds two membrane G-protein-coupled receptors, AT-1 and AT-2 receptors. The majority of the negative haemodynamic (vasoconstriction and hypertension) and cellular (collagen synthesis, collagen deposition and cytokine production) [14–16] effects of Ang II is mediated by the renin/ACE/Ang II–AT-1 receptor axis, the well-established target of ACE-inhibitors and angiotensin type 1 receptor blockers. On the contrary, it has been shown that the activation of the angiotensin type-2 (AT-2) receptors can counterbalance some of the actions mediated by AT-1 receptors, resulting in a significant organ protection [17–19].

Compound 21 (C21), a nonpeptide, highly selective AT-2 receptor agonist [20], directly stimulates the AT-2 receptors, thus representing an important new tool to investigate the AT-2 receptor-mediated effects.

Increasing data demonstrate that C21 has positive effects in the cardiovascular system, it actually improves the cardiac function in rat with myocardial infarction [21,22], and reduces the cardiovascular fibrosis both in stroke-prone spontaneously hypertensive rats [23], and in an experimental model of Nω-nitro-l-arginine-methyl ester-induced hypertension [24].

In the kidney, C21 administration blunts renal inflammatory cell infiltration in renovascular hypertension [25] and in stroke prone spontaneously hypertensive rats [23,26], showing nephroprotective effects in obese Zucker rats [27,28] and in diabetic nephropathy [29,30].

The aim of the present study was to investigate the effect of C21 administration on CsA nephropathy. The effect of C21 administration on the myocardium in CsA-dependent hypertension was also evaluated.

## MATERIALS AND METHODS

### Experimental protocol

Animal husbandry was in conformity with the Institutional Guidelines in compliance with National laws and policies (D.L.n. 116, Gazzetta Ufficiale della Repubblica Italiana, suppl. 40, Feb. 18, 1992) and experiments were performed in accordance with the Guide for the Care and Use of Laboratory Animals published by the US National Institutes of Health (NIH Publication No. 85-23, revised 1996).

Conscious male Sprague–Dawley rats, 150–200 g body weight (BW; Charles River), were individually housed in cages (or metabolic cages as necessary to collect 24 h urine samples) in a temperature-controlled room (22°C) with a 12:12 light–dark cycle and free access to low-salt diet (Teklad 7034) and tap water. Low-salt diet was used to increase renal cyclosporine damage [31,32].

BW (g) and systolic blood pressure (SBP, mmHg) were measured at the beginning and weekly during the experimental period by the tail cuff method (average of six recordings. BP Recorder, Ugo Basile Instruments) by an investigator who was unaware of the specific treatments.

To induce nephropathy, cyclosporine-A (Sandimmun, Novartis) was administered at the dose of 15 mg/kg per day by intraperitoneal injection for 8 days (short-term treatment) and for 28 days (long-term treatment). C21 (Vicore Pharma) was administered at the dose of 0.3 mg/kg per day by intraperitoneal injection. The dose used was chosen based on the demonstration of efficacy on tissue remodelling in previous experimental studies [19,21,24,25,29]. To evaluate the effects of short-term treatment (8 days) a group of 18 rats were studied (control, *n*=5; CsA-treated rats, *n*=6; CsA+C21-treated rats, *n*=7). A group of 24 rats (control, *n*=8; CsA-treated rats, *n*=8; CsA+C21 treated-rats, *n*=8) were studied to evaluate the effects of long-term treatment (28 days). At the end of the experimental periods, blood samples were collected to measure creatinine (Cobas Mira Plus Instrument, Roche), then the rats were killed with an overdose of anaesthesia (sodium pentobarbital). Urinary albumin excretion was measured at the beginning and at the end of experimental periods, on 24 h urine collections by Elisa (Nephrat II Elisa, Exocell). After the sacrifice, kidneys and hearts were immediately excised and weighted. Kidneys transmural sections were fixed with 10% formalin, embedded in paraffin and used for light microscopic examination, morphometric analysis of glomerular, tubulo-interstitial and perivascular fibrosis and monocyte/macrophage, type I and type IV collagen immunostaining. The apex of the heart was cut, frozen in liquid nitrogen and stored at −80°C until RNA extraction. The heart was sectioned into three transverse slices from the apex to the base, fixed with 10% formalin, embedded in paraffin and used for light microscopic examination and morphometric analysis of interstitial and perivascular collagen and monocyte/macrophage immunostaining.

### Histological analysis and morphometric evaluation of renal and myocardial fibrosis

For all rats renal transmural sections (4 μm) and coronal cardiac sections (4 μm) were deparaffinized, rehydrated and stained with haematoxylin–eosin (H&E), following standard techniques. Changes in renal and cardiac morphology were assessed by light microscopic analysis of sections. For all rats a renal consecutive section (4 μm) and a coronal cardiac section (4 μm) were also stained with collagen-specific sirius red and used for morphometric analysis of renal and myocardial fibrosis, as previously described [33,34]. Briefly, slides were examined with a Leica microscope (Leitz Camera) using normal light. A computerized digital camera (Olympus Camedia 5050, Olympus) was used to capture 5 Mp (24-bit colour depth) images (stored as JPG files) and analysed for renal collagen volume fraction in glomerular, tubulo-interstitial and perivascular areas and in interstitial and perivascular cardiac areas with a computerized imaging software (ImageJ, NIH). Two pathologists, blinded to the experimental protocol, performed image analysis.

Glomerular and tubulo-interstitial collagen volume fractions were evaluated at ×200 magnification, respectively, in semi-automated fashion in 10 traced glomeruli (considered as matrix, cells, capillary loops and space surrounding glomerular segments) and in automated fashion in 10 fields without vessels or glomeruli, randomly selected from each kidney section.

The glomerular collagen fraction was expressed as the ratio between red-stained collagen area and glomerular area; the tubulo-interstitial collagen fraction was expressed as the ratio between collagen area and total area.

For analysis of perivascular fibrosis, 10 vessels were randomly selected at ×400 magnification from each kidney section. The image analysis of intraparenchimal vessels was performed in semi-automated fashion. Only the collagen immediately surrounding each intraparenchimal vessel was considered to represent perivascular collagen deposition. The perivascular collagen volume fraction was expressed as the ratio between collagen area surrounding the traced vessel and total cross-sectional area, in order to correct differences in vessel size.

Myocardial total collagen volume fraction was measured on 10 randomly selected microscopic fields of the left ventricular (LV) coronal slice (×200 magnification) and was automatically calculated as the ratio between red-stained interstitial area and the total area of the heart section. For analysis of the perivascular collagen volume fraction, five randomly selected vessels from LV coronal slice were traced and semi-automatically measured (×400 magnification). The collagen immediately surrounding each intramyocardial vessel was considered to represent perivascular collagen deposition and expressed as the ratio between perivascular collagen area and luminal media area [33].

### Immunohistochemical evaluation of monocyte/macrophage renal and myocardial infiltration

The evaluation of monocytes/macrophages infiltration was performed on formalin fixed and paraffin embedded renal and heart sections (3 μm) using a monoclonal mouse anti-rat monocytes/macrophage (CD68, Chemicon), as previously described [29]. The sections were deparaffinized and rehydrated, treated by boiling in citrate buffer (0.01 mol/l, pH 6) in microwave (750 W), and incubated over night at 4°C with primary antibody (1:300). Ultra Tek HRP (Anti-Mouse) Staining System (Scy TeK Laboratories) was used to label the primary antibody. The reaction product was visualized with 3,3’-diaminobenzidine (DAB) (Dako). Negative control was obtained by omitting the primary antibody. Sections were viewed using a Leica microscope (Leitz Camera), and digital images were taken using a computerized digital camera (Olympus Camedia 5050, Olympus). Two independent pathologists blinded to the treatment counted the positive cells in 10 randomly selected non-overlapping fields per section at ×200 magnification and took the average. The macrophages were expressed as number of positive cells/fields [29].

### Type I collagen and type IV collagen immunohistochemistry

Immunohistochemical evaluation was performed on consecutive sections (3 μm) of formalin fixed, paraffin embedded renal tissue. Sections were deparaffinized in xylene and rehydrated through graded alcohol series. Endogenous peroxidase activity was blocked by 3% hydrogen peroxide. The sections were treated with microwave (pH 6 citrate buffer). The sections were then incubated at 4°C overnight with type I collagen (1:300, rabbit anti-rat polyclonal antibody, Millipore Chemicon, AB755P) or type IV collagen (1:150, rabbit anti-human polyclonal antibody, Abcam, AB6586). The reaction was amplified with LSAB2+System-HRP, as previously described [9]. A positive immunoreaction was identified after incubation with DAB and counterstaining with Mayer haematoxylin. Negative controls were obtained omitting the primary antibody. Sections were viewed using a Leica microscope (Leitz Camera). Two independent pathologists, blinded to the treatment, analysed the immunostaining with a Leica microscope (Leitz Camera) and, subsequently, for each rat, 5-Mp (24-bit colour depth) images of glomerular and tubulo-interstitial area (20× objective) were captured by a computerized digital camera (Olympus Camedia 5050, Olympus). Immunostaining was quantified by a pathologist, using a computerized imaging software (ImageJ, NIH), respectively, in semi-automated fashion in 10 traced glomeruli and in automated fashion in 10 tubulo-interstitial fields (without vessels or glomeruli), randomly selected from each kidney section. Type 1 and type IV collagen immunostaining was expressed as percentage (ratio of immunostaining area to total area or glomerular area) [35].

### Myocardial collagen analysis by polarized light microscope

Sirius Red-stained myocardial coronal section was also analysed in the long-term protocol using a light microscope under polarized light to evaluate the different types of collagen in interstitial and perivascular areas. By polarized light microscope, collagen type I appears yellow/orange, whereas collagen type III presents as green [36]. For each rat, randomly selected microscopic fields of interstitial and perivascular areas were analysed with polarized light microscope using a 20× objective (Leiz Camera). Images were captured by a computerized digital camera (Olympus Camedia 5050, Olympus) and analysed for different type of collagen by the same pathologists, as previously described [33].

### Total RNA extraction, reverse transcription and myocardial Col1A1 and fibronectin mRNA expression

Total RNA was extracted from the heart using Mirvana Kit (Ambion, Lifetechnologies), following the manufacturer's instructions. One microgram of RNA was reverse-transcribed to synthesize cDNA (High Capacity cDNA Reverse Transcription kit, Applied Biosystems).

Myocardial mRNA expression of collagen 1A1 (Col1A1), fibronectin (FN-1) and glyceraldehyde-3-phosphate dehydrogenase (GAPDH), used as reference gene, was evaluated by real-time PCR using the TaqMan Realtime ABI Prism 7900 HT Sequence Detection System (Applied Biosystems). Col1A1, FN-1 and GAPDH mRNA expression were evaluated using the Assay-on-Demand Gene Expression Product (Applied Biosystems), following the manufacturer's instructions. All reactions were performed in duplicate. Expression levels were normalized to GAPDH mRNA expression, following the 2^−^ΔΔCt [35].

### Statistical analysis

Data are presented as means ± S.E.M. Differences among the groups of rats (control, CsA and CsA+C21-treated rats) for SBP, BW, kidney/BW, heart/BW, glomerular filtration rate (GFR), 24 h urinary albumin excretion, renal glomerular, tubulo-interstitial and perivascular fibrosis, myocardial interstitial and perivascular fibrosis, renal inflammatory infiltrates and type I and type IV collagen expression, and myocardial Col1A1 and FN-1 mRNA expression were assessed with the use of ANOVA followed by the Fisher's protected least-significant test for post-hoc comparisons. Differences between means were considered significant at *P*<0.05.

## RESULTS

### Short-term protocol

#### Effects of chronic short-term C21 treatment on systolic blood pressure, glomerular filtration rate and urinary albumin excretion in CsA-treated rats

Table 1 shows the effects of chronic short-term C21 administration on SBP, BW, kidney/BW, heart/BW, GFR and urinary albumin excretion in CsA-treated rats.

**Table 1:** SBP (mmHg), BW (g), kidney/BW ratio (mg/g), heart/BW ratio (mg/g), GFR (ml/min), urinary albumin excretion (UAE, mg/24 h) in control, CsA and CsA+C21-treated rats at the end of 8 days of treatment *P<0.01 compared with control group.

	Control	CsA	CsA+C21
SPB, mmHg	140.1±2.2	176.2±2.8*	174.2±2.5*
BW, g	384.75±13.48	335.16±5.51*	341.71±8.42*
Kidney/BW, mg/g	3.905±0.169	3.879±0.098	4.005±0.089
Heart/BW, mg/g	3.192±0.116	2.958±0.073	3.194±0.075
GFR, ml/min	1.716±0.241	1.681±0.248	1.663±0.262
UAE, mg/24 h	1.53±0.62	1.89±0.30	1.58±0.73

After 8 days of treatment, SBP was significantly higher in the CsA-treated rats and CsA+C21-treated rats compared with the control animals. C21 did not modify the increase of blood pressure in CsA-treated rats.

BW was significantly reduced in CsA-treated and CsA+C21-treated rats with respect to control rats (Table 1). Kidney weight/BW ratio and heart weight/BW ratio were similar among the groups (Table 1). CsA and C21 treatment for 8 days did not modify GFR and urinary albumin excretion as compared with control rats (Table 1).

#### Effects of chronic short-term C21 treatment on renal fibrosis and inflammatory cell infiltration

At the end of the experimental period, histologic examination showed focal dilatation of cortical tubules and a greater number of interstitial monocytic cells in CsA and CsA+C21 treated rats with respect to control rats.

**Figure 1:**
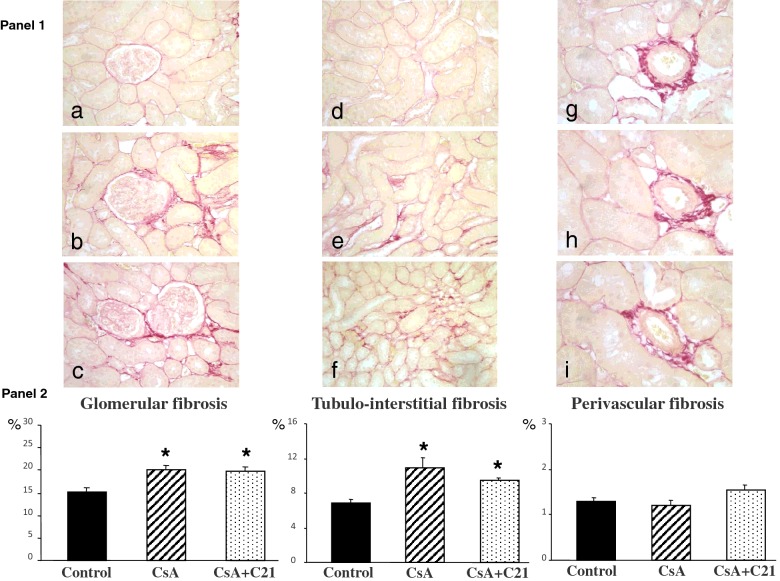
Effect of C21 administration for 8 days on renal fibrosis in CsA-treated rats Panel 1: Representative photomicrographs of glomerular, tubulo-interstitial and perivascular fibrosis in control (**a**, **d**, **g**), CsA (**b**, **e**, **h**), CsA+C21-treated (**c**, **f**, **i**) rats (sirius red staining: glomerular and tubulo-interstitial fields, ×20; perivascular fields, ×40). Staining showed an increase in glomerular and tubulo-interstitial fibrosis in CsA-treated rats (**b** and **e**) as compared with control rats (**a** and **d**). C21 treatment (**c** and **f**) did not modify glomerular and tubulo-interstitial fibrosis in CsA-treated rats. Perivascular fibrosis did not change both in CsA-treated rats (**h**) and CsA+C21-treated rats (**i**). Panel 2: Quantification of glomerular, tubulo-interstitial and perivascular fibrosis in the different groups of rats. Data are means ± S.E.M. *P<0.01 compared with control group.

The morphometric analysis showed a significant increase in glomerular and tubulo-interstitial fibrosis, quantified by computerized image analysis after sirius red staining, in CsA-treated rats, whereas perivascular fibrosis was not modified (Figure 1, Panels 1 and 2). Administration of C21 for 8 days did not modify the increase in glomerular and tubulo-interstitial fibrosis in CsA-treated rats (Figure 1, Panels 1 and 2).

**Figure 2:**
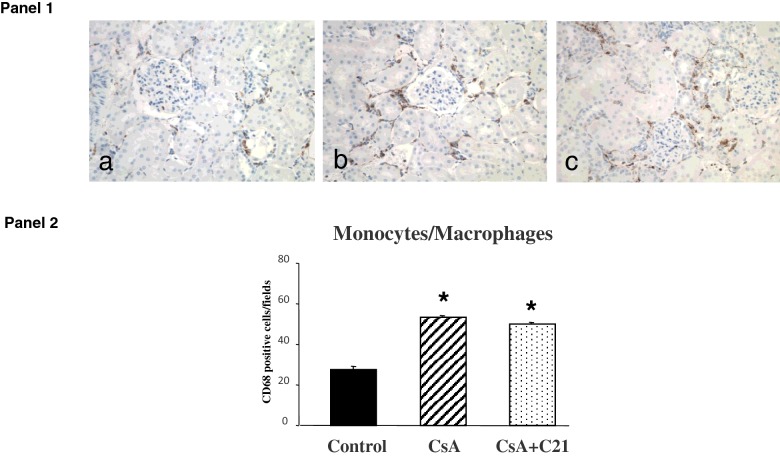
Effects of C21 administration for 8 days on renal inflammatory cell infiltration in CsA-treated rats Panel 1: Immunohistochemical identification of renal inflammatory cell infiltration in control (**a**), CsA-treated rats (**b**) and CsA+C21-treated rats (**c**) (original magnification ×20). The immunostaining showed a significant increase in interstitial monocyte/macrophage cells (brown reaction) in CsA-treated rats (**b**) as compared with control rats (**a**). C21 administration did not modify the increase of monocyte/macrophage infiltration in CsA-treated rats (**c**). Panel 2: Quantification of staining of renal monocyte/macrophage inflammatory cells in the different groups of rats. Data are means ± S.E.M. *P<0.01 compared with control group.

Similarly to what observed for the fibrosis, CsA administration caused a significant increase in monocyte/macrophage infiltration in renal tissue when compared with control rats (Figure 2, Panels 1 and 2). C21 administration did not modify the increase in monocyte/macrophage infiltration caused by CsA treatment (Figure 2, Panels 1 and 2).

#### Effects of chronic short-term C21 treatment on myocardial fibrosis and inflammatory cell infiltration

**Figure 3:**
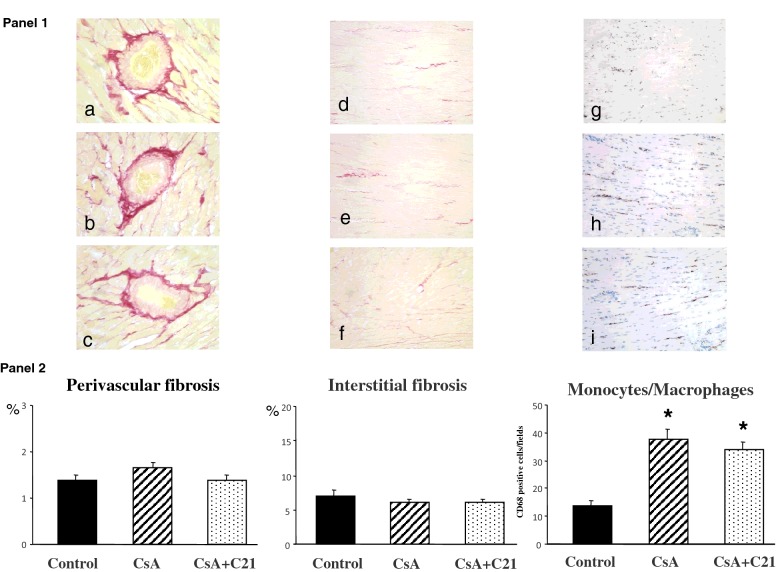
Effects of C21 administration for 8 days on myocardial fibrosis in CsA-treated rats Panel 1: Representative photomicrographs of perivascular and interstitial fibrosis and inflammatory cell infiltration in left ventricle of control (**a** and **d**), CsA-treated (**b** and **e**) and CsA+C21-treated rats (**c** and **f**). Sirius red staining: interstitial fields, original magnification ×20; perivascular fields, original magnification ×40. Sirius red staining did not show changes in myocardial fibrosis, both at perivascular and interstitial area in CsA-treated rats (**b** and **e**) and CsA+C21-treated rats (**c** and **f**). Immunohistochemical staining of myocardial inflammatory cell infiltration showed a significant increase in interstitial monocyte/macrophage cells in CsA-treated rats (**h**) compared with control rats (**g**). C21 administration did not modify the increase of monocyte/macrophage infiltration in CsA-treated rats (**i**). Panel 2: Quantification of myocardial perivascular and interstitial fibrosis and monocyte/macrophage inflammatory cells in the different groups of rats. Data are means ± S.E.M. *P<0.01 compared the control group.

The evaluation of myocardial fibrosis after sirius red staining is shown in Figure 3. Both CsA treatment and C21 administration for 8 days did not significantly modify myocardial fibrosis both at perivascular and interstitial area (Figure 3, Panels 1 and 2). Differently, CsA administration caused a significant increase in monocyte/macrophage infiltration in myocardial tissue when compared with control rats (Figure 3, Panels 1 and 2). C21 administration did not modify the increase in monocyte/macrophage infiltration caused by CsA treatment (Figure 3, Panels 1 and 2).

### Long-term protocol

#### Effects of chronic long-term C21 treatment on systolic blood pressure, glomerular filtration rate and urinary albumin excretion in CsA-treated rats

**Figure 4:**
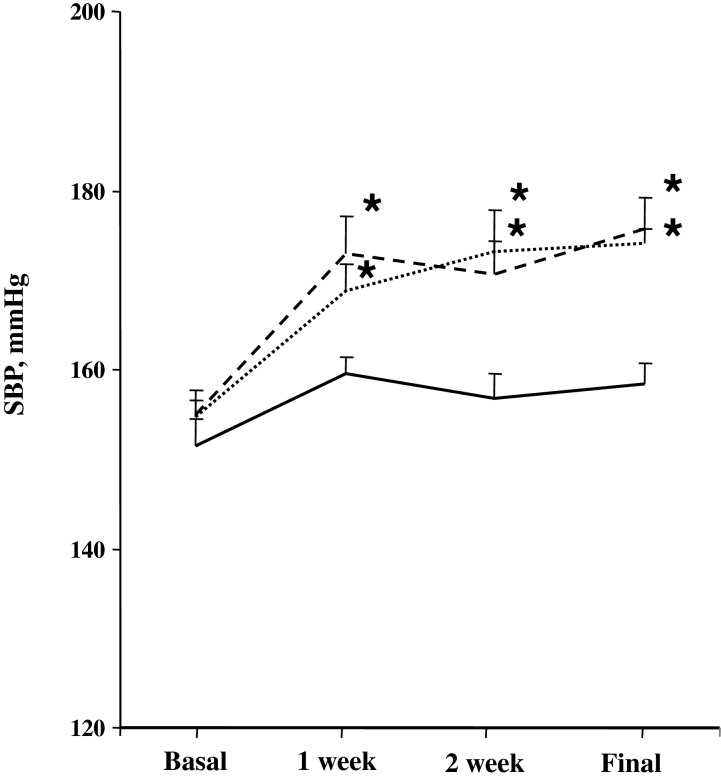
Time course of blood pressure in CsA nephropathy before and during C21 administration Control, continuous line; CsA, dotted line; CsA+C21-treated rats, dashed line. *P<0.01 compared with corresponding basal values and control group.

Figure 4 shows the effects of chronic long-term C21 administration on SBP during the experimental period. SBP was significantly higher in the CsA-treated rats and CsA+C21-treated rats compared with the control animals during the entire experimental period, starting from the first week of the treatment. C21 did not modify the increase of blood pressure in CsA-treated rats (Figure 4)

Table 2 shows the effects of chronic long-term C21 administration on BW, kidney/BW, heart/BW, GFR and urinary albumin excretion in CsA-treated rats.

**Table 2:** BW (g), kidney/BW ratio (mg/g), heart/BW ratio (mg/g), GFR (ml/min), urinary albumin excretion (UAE, mg/24 h) in control, CsA, and CsA+C21-treated rats at the end of 28 days of treatment *P<0.01 compared with control group.

	Control	CsA	CsA+C21
BW, g	401.42±2.02	368.65±8.58*	360.66±7.52*
Kidney/BW, mg/g	3.929±0.145	3.902±0.108	4.130±0.083
Heart/BW, mg/g	2.927±0.134	2.942±0.048	3.129±0.121
GFR, ml/min	2.788±0.899	2.040±0.290	2.929±0.624
UAE, mg/24 h	2.13±0.45	2.65±0.42	2.08±0.23

BW was significantly reduced in CsA-treated and CsA+C21-treated rats with respect to control rats (Table 2). Kidney weight/BW ratio and heart weight/BW ratio were similar among the groups. (Table 2). GFR and urinary albumin excretion did not show significant changes among the groups, even if a slight decrease in GFR in CsA-treated rats was observed (Table 2).

#### Effects of chronic long-term C21 treatment on renal fibrosis, type I and type IV collagen, and inflammatory cell infiltration

In the kidney, morphologic examination showed dilated cortical tubules and the presence of interstitial inflammatory cells in CsA and CsA+C21-treated rats.

**Figure 5:**
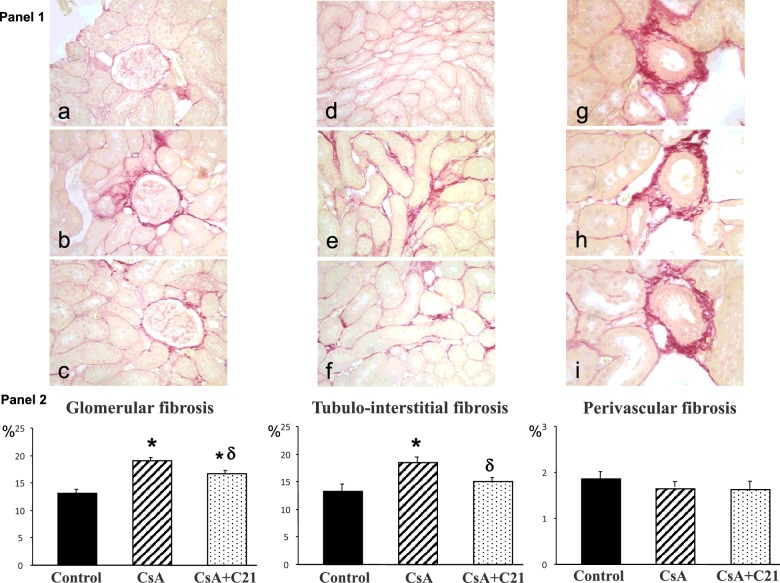
Effect of C21 administration for 28 days on renal fibrosis in CsA-treated rats Panel 1: Representative microscopic images of glomerular, tubulo-interstitial and perivascular fibrosis in control (**a**, **d**, **g**), CsA (**b**, **e**, **h**), CsA+C21-treated (**c**, **f**, **i**) rats (sirius red staining: glomerular and tubulo-interstitial fields, original magnification ×20; perivascular fields, original magnification ×40). Staining showed an increase in glomerular and tubulo-interstitial fibrosis in CsA-treated rats (**b** and **e**) as compared with control rats (**a** and **d**). C21 treatment (**c** and **f**) reduced glomerular and tubulo-interstitial fibrosis in CsA-treated rats. Perivascular fibrosis did not change both in CsA-treated rats (**h**) and CsA+C21-treated rats (**i**). Panel 2: Quantification of glomerular, tubulo-interstitial and perivascular fibrosis in the different groups of rats. Data are means ± S.E.M. *P<0.01 compared with control group; ^δ^*P*<0.01 compared with CsA-treated rats

**Figure 6:**
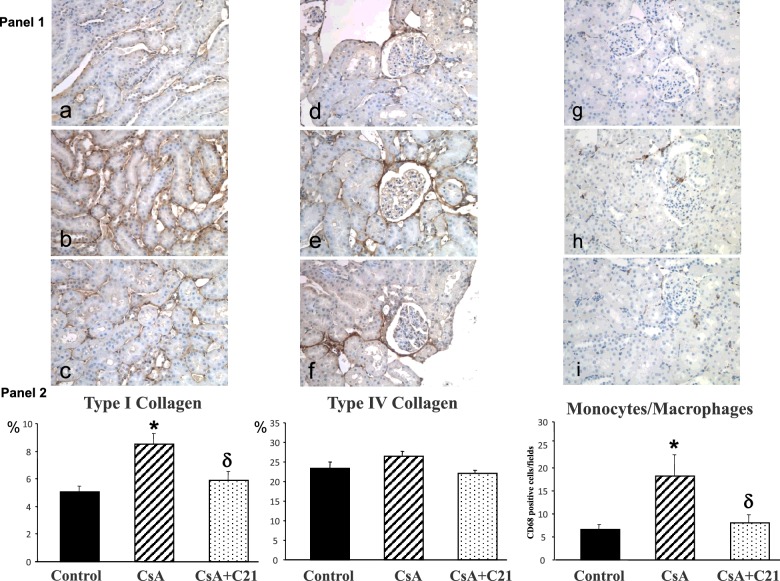
Effects of C21 administration for 28 days on type I and type IV collagen and inflammatory cell infiltration in the kidney of CsA-treated rats Panel 1: Representative photomicrographs of immunohistochemical characterization of renal type I and type IV collagen and inflammatory cell infiltration in control (**a**, **d**, **g**), CsA-treated rats (**b**, **e**, **h**), and CsA+C21-treated rats (**c**, **f**, **i**). The immunostaining (brown reaction, 20×) showed a significant increase in interstitial type I collagen in CsA-treated rats (**b**) compared with control rats (**a**). C21 administration reduced the increase of type I collagen in CsA-treated rats (**c**). Type IV collagen in glomerular area did not modify in CsA (**e**) and CsA+C21-treated (**f**) rats respect to control rats (**d**). CsA treatment caused an increase in monocyte/macrophage cell infiltration in CsA-treated rats (**h**) as compared with control rats (**g**). C21 administration reduced the increase of monocyte/macrophage cell infiltration in CsA-treated rats (**i**). Panel 2: Quantification of staining of renal type I collagen, type IV collagen and monocyte/macrophage inflammatory cells in the different groups of rats. Data are means ± S.E.M. *P<0.01 compared with control rats, ^δ^*P*<0.01 compared with CsA-treated rats.

Glomerular and tubulo-interstitial fibrosis were significantly increased in CsA-treated rats (Figure 5, Panels 1 and 2), whereas perivascular fibrosis was not modified. Administration of C21 for 28 days blunted the increase in glomerular fibrosis in CsA-treated rats, which remained higher compared with control rats (Figure 5, Panels 1 and 2). Administration of C21 for 28 days caused a significant reduction in tubulo-interstitial fibrosis in CsA-treated rats (Figure 5, Panels 1 and 2), toward values similar to control rats. CsA administration caused a significant increase in type I collagen at tubulo-interstitial level, blocked by C21 treatment, whereas type IV collagen at glomerular level did not significantly changed (Figure 6, Panels 1 and 2).

CsA administration caused a significant increase in monocyte/macrophage infiltration in renal parenchyma, localized in the tubulo-interstitial area, when compared with control rats (Figure 6, Panels 1 and 2). C21 administration completely blocked inflammatory cell infiltration caused by CsA administration (Figure 6, Panels 1 and 2).

#### Effects of chronic long-term C21 treatment on myocardial fibrosis, type 1 collagen and fibronectin mRNA expression and inflammatory cell infiltration

**Figure 7:**
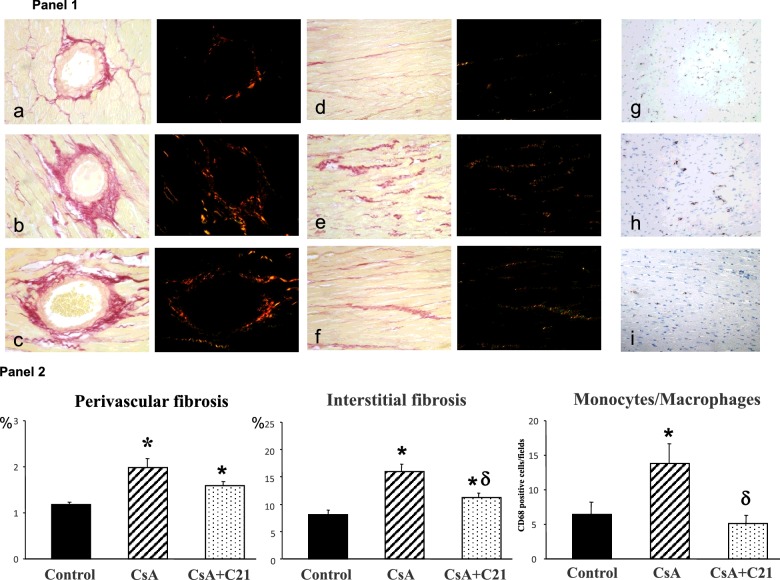
Effects of C21 administration for 28 days on myocardial fibrosis and monocyte/macrophage cell infiltration in CsA-treated rats Panel 1: Representative photomicrographs of the LV perivascular and interstitial fibrosis and inflammatory cell infiltration in control (**a**, **d**, **g**), CsA-treated (**b**, **e**, **h**) and CsA+C21-treated rats (**c**, **f**, **i**). Sirius red staining showed an increase in myocardial fibrosis, both at perivascular (×40) and interstitial (×20) area in CsA-treated rats (**b** and **e**) and CsA+C21-treated rats (**c** and **f**) as compared with control rats (**a** and **d**). C21 treatment caused a significant decrease in interstitial fibrosis (**f**) respect to CsA-treated (**e**) rats. The polarized light analysis showed the prevalence of type I collagen fibres (yellow/orange) in the heart of all the rat groups. Representative photomicrographs of immunohistochemical characterization of myocardial inflammatory cell infiltration (×20) (**g**, **h**, **i**). There was a significant increase in interstitial monocyte/macrophage cells (brown reaction) in CsA-treated rats (**h**) as compared with control rats (**g**). C21 administration reduced the increase of monocyte/macrophage infiltration in CsA-treated rats (**i**), as compared with CsA-treated rats. Panel 2: Quantification of myocardial perivascular and interstitial fibrosis and monocyte/macrophage inflammatory cells in the different groups of rats. Data are means ± S.E.M. *P<0.01 compared with control group. ^δ^*P*<0.01 compared with CsA-treated rats.

Figure 7 shows the evaluation of myocardial fibrosis after sirius red staining with corresponding analysis of collagen fibres by polarized light microscope. CsA treatment for 28 days significantly increased myocardial fibrosis both at perivascular and interstitial area (Figure 7, Panels 1 and 2). C21 administration blunted the development of myocardial fibrosis in myocardial tissue, mainly at interstitial level (Figure 7, Panels 1 and 2). The analysis with polarized light microscope shows that perivascular and interstitial fibrosis (Figure 7, Panel 1) is due to a prevalent increase of type I collagen (orange stain). CsA administration caused a significant increase in monocyte/macrophage infiltration in myocardial tissue when compared with control rats (Figure 7, Panels 1 and 2). C21 administration blocked the increase in monocyte/macrophage infiltration caused by CsA treatment (Figure 7, Panels 1 and 2).

**Figure 8:**
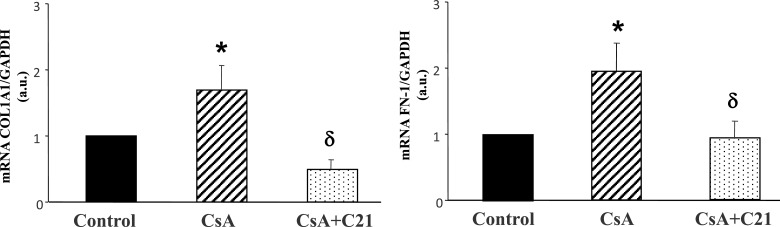
Effect of CsA and C21 administration on collagen 1A1 (Col1A1) and fibronectin (FN-1) mRNA expression in the heart by real-time PCR Data are means ± S.E.M. *P<0.01 compared with control group. ^δ^*P*<0.01 compared with CsA-treated rats.

In the hearts of CsA-treated rats Col1A1 and FN-1 mRNA expression, evaluated by quantitative real time PCR, were significantly increased as compared with control rats (Figure 8). C21 administration completely blocked the increase in Col1A1 and FN-1 mRNA expression in the hearts of CsA-treated rats (Figure 8).

## DISCUSSION

The results of the present study demonstrate that chronic C21 treatment promotes nephroprotection in CsA nephropathy, by reducing renal fibrosis and inflammatory cell infiltration, in absence of blood pressure modifications.

CsA administration, after both 1 and 4 weeks, caused an increase in blood pressure and renal fibrosis at glomerular and tubulo-interstitial area, but not at perivascular level, and an increase in inflammatory cell infiltration. C21 treatment for 1 week did not modify the increase of blood pressure caused by CsA administration and did not show any effect on glomerular and tubulo-interstitial fibrosis and monocyte-macrophage infiltration.

After 4 weeks, C21 treatment did not modify the increase of blood pressure caused by CsA administration, whereas reduced glomerular and tubulo-interstitial fibrosis and blocked inflammatory cell infiltration.

CsA nephropathy is predominantly characterized by tubulo-interstitial fibrosis, with evidence of fibrotic stripes in the interstitial space and tubular damage [8].

Our data confirm the characteristics of these lesions, but using computerized image quantification techniques, we were able to detect the presence of fibrosis also in the glomerular area, although of lesser degree respect to the tubulo-interstitium. After 4 weeks of treatment, the antifibrotic effect of C21 is also present at the glomerular area, but it is stronger at tubulo-interstitial level, where not only significantly reduces fibrosis compared with the CsA-treated group, but also brings to values not different compared with the control group.

The administration of CsA caused an increase in inflammatory infiltrates, which is predominantly localized at tubulo-interstitial level. C21 treatment for 4 weeks markedly reduced the inflammatory cell infiltration, supporting the notion that the antifibrotic effect of C21 is mediated by an anti-inflammatory action.

As occurs also in patients [11], CsA treatment caused an increase in blood pressure that was not changed by C21 administration. Furthermore, the C21 antifibrotic effect, observed in the kidney, is independent on changes in blood pressure level.

At difference with the kidney, the development of cardiac fibrosis appears to be delayed. In fact, myocardial fibrosis, both at perivascular and interstitial level, was not observed after 1 week of CsA administration, when inflammatory cell infiltrates were already present, a dysfunction that persisted after 4 weeks.

After 4 weeks, CsA caused a significant increase in myocardial fibrosis, both at perivascular and interstitial level, associated with an increase of inflammatory infiltrates.

At difference with the kidney, where perivascular fibrosis was undetectable, in the heart perivascular fibrosis was stronger than interstitial fibrosis after CsA administration.

In CsA-treated rats C21 significantly reduced myocardial interstitial fibrosis, blocking the inflammatory cell infiltration, but did not significantly reduce perivascular fibrosis, even if it was a trend to a reduction.

Although there are common features between the renal and cardiac fibrosis, our findings support the hypothesis that differences exist not only between the two different organs, but also among different districts of the same organ.

The findings that in our experimental conditions we did not observe perivascular fibrosis in the kidney, that was instead present at myocardial level, may depend on the increase in blood pressure caused by CsA administration, that is perceived more by myocardial intraparenchymal vessels, whereas the renal intraparenchymal arterioles are better protected.

In addition, the findings that in both kidney and heart the antifibrotic action of C21 is major at the interstitial level, where inflammatory infiltrates are concentrated, further suggests that the antifibrotic action of C21 is mediated by an anti-inflammatory mechanisms. In fact, the decrease of tissue fibrosis is present only in the long-term treatment with C21, when a complete reduction of the inflammatory cell infiltration has been achieved, whereas it is not evident in the short-term treatment, in the presence of still high inflammatory cell infiltrates.

In summary, in the present study we have shown that stimulation of the protective arm of the renin angiotensin system, through the selective agonist of AT-2 receptors C21, counteract tubulo-interstitial fibrosis, the most potent predictor of the progression of renal diseases.

## Abbreviations

ACE: angiotensin converting enzyme
Ang II: angiotensin II
AT-2: angiotensin type-2
BW: body weight
C21: compound 21
Col1A1: collagen 1A1
CsA: cyclosporine-A
DAB: 3,3’-diaminobenzidine
FN-1: fibronectin
GFR: glomerular filtration rate
LV: left ventricular
SBP: systolic blood pressure
